# Evaluating the structure and process of effective integrated care for people with multiple long-term conditions: systematic review protocol

**DOI:** 10.1136/bmjopen-2026-116356

**Published:** 2026-06-23

**Authors:** Olamide Todowede, Sarah Damery, Naila Dracup, Jeremiah Donoghue, Furqan Butt, Amy Grove, Arabella Scantlebury

**Affiliations:** 1 Centre for Evidence and Implementation Science, University of Birmingham, Birmingham, UK

**Keywords:** Multimorbidity, Delivery of Health Care, Integrated, Chronic Disease

## Abstract

**Abstract:**

**Introduction:**

Integrated care represents a transformative approach to delivering person-centred healthcare, aiming to reduce health inequalities, improve outcomes and boost system efficiency. The WHO advocates integrated care, which is also a central pillar of the UK government’s National Health Service (NHS) 10-year plan. However, there remains a significant evidence gap regarding optimal organisational strategies for designing and delivering integrated care services. Multiple long-term conditions (MLTCs) refer to the coexistence of two or more chronic physical and/or mental health conditions in a person. This complex need requires coordinated support from multiple healthcare services, and individuals with MLTCs may particularly benefit from integrated care services. This review aims to identify and synthesise evidence regarding the organisational structures (care set-up) and processes (care delivery) that support integrated care models for adults with MLTCs.

**Methods:**

An information specialist and research team will co-develop a search strategy and search databases (MEDLINE, EMBASE and PsycINFO) for empirical articles between January 1990 and December 2025. Date restrictions reflect the establishment of integrated care as a concept for organising and delivering health services. Screening, data extraction and methodological assessment using the Mixed Methods Appraisal Tool will be independently conducted by two researchers. Eligible studies include any empirical studies, including all study designs that investigate adults (aged 18+ years) with MLTCs in high-income countries. This review aims to identify the way integrated care services are designed and delivered. As such, studies must identify and explain the organisational structures and processes involved in implementing and delivering integrated care services to be eligible for inclusion. Extracted data will be categorised according to the two integrated care mechanisms: vertical and horizontal integration. Data will then be examined in relation to how these mechanisms function and/or interact across the macro (system-wide structures and policies), meso (organisational arrangements) and micro (clinical practice and patient-level interactions) levels of integrated care. Review findings will be reported narratively.

**Ethics and dissemination:**

Ethical approval is not required. This work may directly inform health policy by providing evidence-based understanding of how to organise and deliver integrated care for people living with MLTCs. The findings will be disseminated through publication in a peer-reviewed journal and shared with relevant stakeholders.

**PROSPERO registration number:**

CRD420251143298.

**STRENGTHS AND LIMITATIONS OF THIS STUDY**

- This review is the first, to our knowledge, to synthesise evidence on the structure and delivery processes of integrated care services for people with multiple long-term conditions (MLTCs).
- The review includes any empirical study design, provided that integrated care interventions have been implemented.
- This study evaluates the structure and processes of integrated care for MLTCs, but does not assess intervention effectiveness, which could be considered for future research.
- To match the UK context, the review includes only studies from World Bank-defined high-income countries. Research on low- and middle-income countries is still needed.

## Introduction

 Globally, healthcare systems are experiencing increasing demand while simultaneously facing pressure to reduce resource use.^1 2^ This is partly driven by increased life expectancy, which has changed disease patterns and means more people are living with multiple long-term conditions (MLTCs).^1^ Also known as ‘multimorbidity’, MLTCs refer to the coexistence of two or more long-term physical and/or mental health conditions in an individual.^3^ In England, one in four adults lives with MLTCs, which places significant pressure on the National Health Service (NHS) due to these individuals having both a higher volume and greater complexity of care needs.^4^

Accessing healthcare is a particular challenge for individuals with MLTCs, who often face a range of challenges engaging with a health system that is fragmented and typically designed to treat single conditions.^5^,^7^ At an individual level, evidence on patient experience of treatment for MLTCs consistently identifies a lack of integrated, patient-centred care, leaving patients feeling that their needs are not fully understood or addressed.^8^ Evidence from healthcare professionals has repeatedly identified communication difficulties, unclear care pathways, inadequate system support and duplicated efforts across different healthcare systems as consistent challenges in treating patients with MLTCs.^9^ Cumulatively, these challenges manifest as adverse patient outcomes (eg, dissatisfaction with care provision, inappropriate polypharmacy (ie, the prescribing of unnecessary or excessive medications), workforce outcomes (eg, staff burnout) and organisational outcomes (eg, fragmented, inefficient care and resource waste).^10 11^

In the 1990s, integrated care was conceptualised as a concept of organising healthcare by focusing on the needs of patients or populations and addressing the adverse outcomes and experiences of care linked to a fragmented healthcare system.^12^ Integrated care provides a potential solution to improve health outcomes for individuals with MLTCs, assuming that they achieve more appropriate and better quality and cost-effective care.^13^ In 2016, the WHO developed the Integrated People-Centred Health Services framework, recommending that health services should work in a joined-up way to deliver equitable, person-centred and coordinated services across different healthcare settings to improve health outcomes and efficiency of healthcare delivery.^14 15^ The NHS 10-Year Plan, ‘Fit for the Future’, also champions integrated care and proposes that, through this system-level change to the way care is organised and delivered, substantial improvements in population health and reductions in health inequalities could be realised.^16^ Despite being at the forefront of global and domestic health policy, evidence to support the introduction of integrated care is limited.^17^ There is a rapidly emerging evidence base about integrated care models for a wide range of healthcare systems.^18^ These models have the potential to increase patient satisfaction, access to and quality of healthcare, system efficiency and cost-effectiveness.^19^,^30^

However, there is a substantial knowledge gap in understanding how best to organise and deliver integrated care models. Robust evidence on the key components, characteristics, structures, processes and integration’s impacts on system efficiency and outcomes remains scarce, particularly for people with MLTCs.^1725 30^,^32^ Addressing this evidence gap is a priority area for individuals with MLTCs, who require coordinated, multiprofessional and multisector support, yet continue to experience fragmented care pathways and varied health outcomes.^33^,^36^ To achieve the NHS Long Term Plan’s ambitious vision for an integrated health and care service, evidence is urgently needed to inform the translation of this policy into effective system-level change. This systematic review will identify how to organise and deliver integrated care models to improve outcomes for adults with MLTCs. Specifically, the review aims to identify and explore the structures and processes of integrated care models that improve health outcomes and increase health system efficiency for adults with MLTCs. In this context, ‘structures’ refer to how care is set up, such as the way different services and infrastructures are arranged or coordinated. The ‘processes’ refer to how care is delivered, for example, referral and care pathways and communication processes. This review will provide evidence to inform current global and UK health policy. Notably, this review will provide evidence that addresses the current UK government’s long-term plan and could be used to optimise service delivery and reduce resource use. The review also addresses research priority areas, including multiple evidence gaps outlined in the National Institute for Health and Care Research Strategic Framework for MLTCs,^37^ and will provide direct evidence to inform numerous current global and national policy priority areas.^17^

### Research question

What are the organisational structures and processes for integrated care systems that lead to better healthcare outcomes and health system efficiency for adults with MLTCs?

Specific objectives include:

1. To identify and explore the core organisational structures and processes for providing integrated care for adults with MLTCs.
2. To identify the key components of integrated care systems that lead to better healthcare outcomes and health system efficiency for adults with MLTCs.

### Review framework

**Figure 1:**
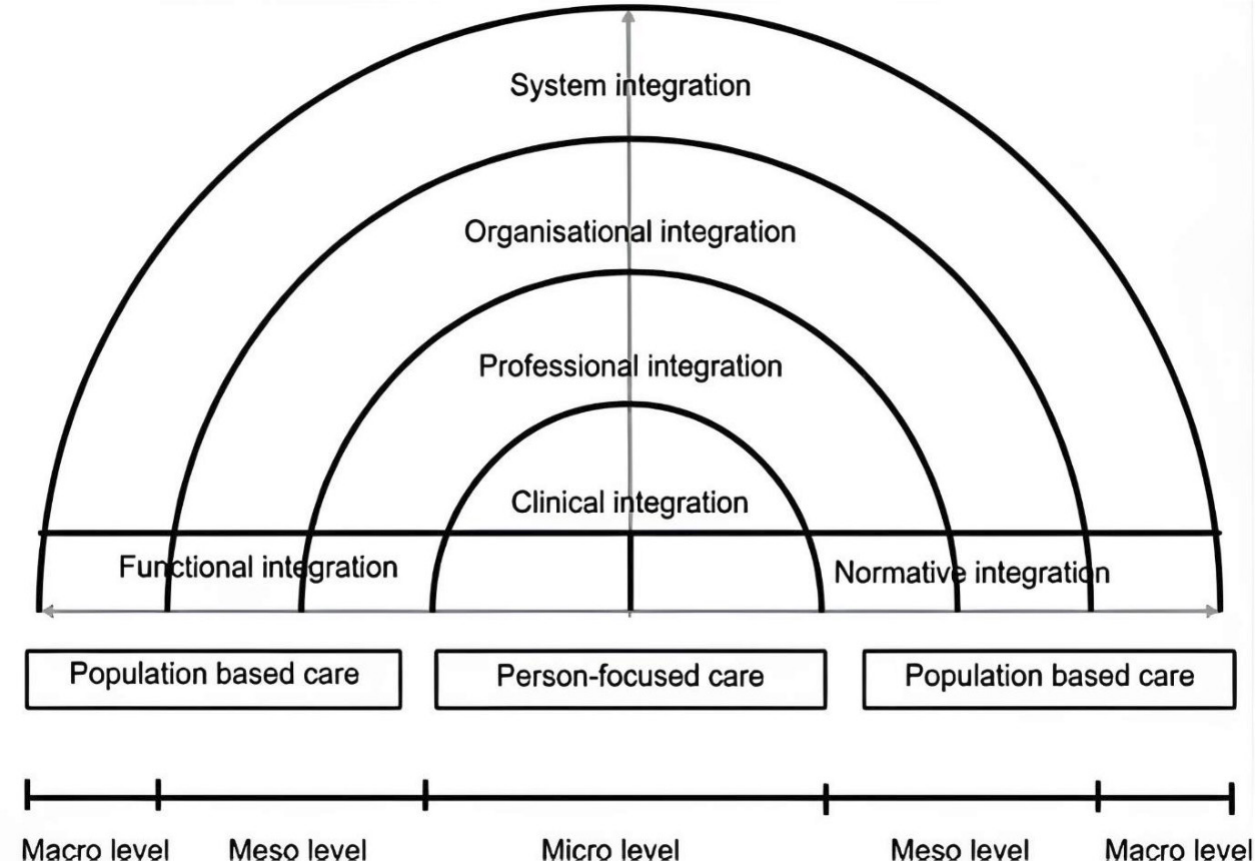
The Rainbow Model of Integrated Care conceptual framework for integrated care.^39^

**Figure 2:**
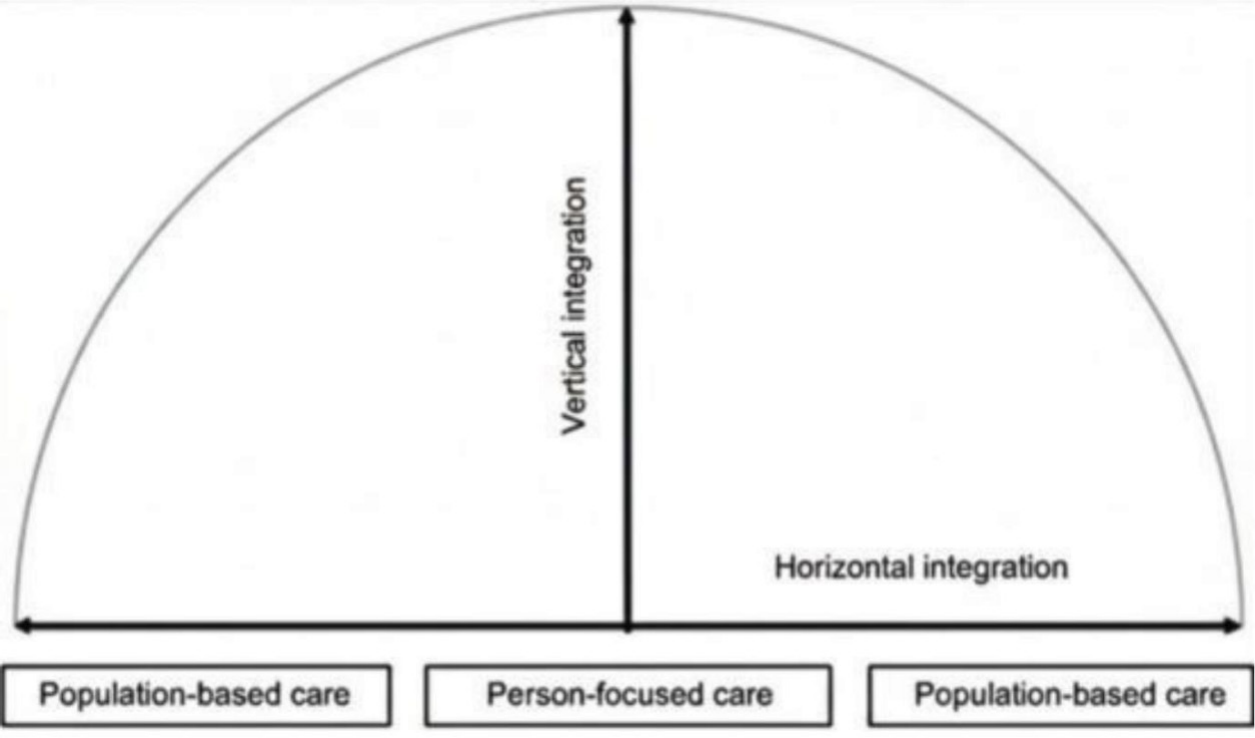
The Rainbow Model of Integrated Care system integration mechanisms.^39^

The Rainbow Model of Integrated Care (RMIC; figure 1) will be used as a framework for characterising the core components of integrated care identified in the included studies.^38 39^ The framework offers a structured approach to understanding how integrated care operates across three levels of the health system: macro (system integration), meso (organisational and professional integration) and micro (clinical, service and personal integration).^39^ The framework will be used to explain how these levels interact through two mechanisms: vertical and horizontal integration (figure 2).^39^ Vertical integration explores how different care settings (eg, primary care and secondary care) work in collaboration to ensure the delivery of efficient and seamless care across the patient pathway, while horizontal integration focuses on understanding systems-level collaboration to facilitate economies of scale and service efficiency (eg, mergers between healthcare organisations or partnerships between health and social care providers).^30 40^

The RMIC framework proposes that both vertical and horizontal integration mechanisms are required to counteract health service fragmentation and that improved health outcomes are achieved by enacting various functional and normative influences (eg, information technology, workforce organisation and communication).^38 39^ The RMIC aims to capture how integrated care actors (such as health professionals, policymakers, service commissioners and patients) plan, coordinate and interact to implement integrated care within and across settings. This framework differs from other health system models that often focus on single-disease management or assess only specific components of integrated care in isolation.^38^ Of particular benefit is the ability to explore all three levels of the health system, which will enable us to identify individual, organisational and system-level levers for change. While we do not anticipate that study authors in our included papers will conceptualise their own approach using the terms within the RMIC framework, we will apply the framework within our analysis to characterise and ‘map’ the structures and processes described within the papers that we assess.

## Methods and analysis

### PROSPERO registration

This protocol is registered on PROSPERO (CRD420251143298) and follows the Preferred Reporting Items for Systematic Review and Meta-Analysis Protocols (PRISMA-P) guideline (online supplemental appendix 3).^41^

### Patient and public involvement

This review is informed by two Patients and Public Involvement contributors (PPI) with lived experience of two or more chronic conditions. They were recruited through the OPTIMising therapies, disease trajectories and AI-assisted clinical management for patients living with complex multimorbidity (OPTIMAL) study at the University of Birmingham.^42^ By providing information about what it is like to live with MLTCs and their personal experiences accessing health services, their contributions have directly informed review planning and protocol development and have identified key terms incorporated into the draft search strategy. As the review progresses, we will conduct a second discussion meeting with the PPI members. Contributors will be directly involved in interpreting our findings and ensuring that our narrative aligns and represents what matters most to patients and their real-world experience. We will be particularly mindful to ensure that any recommendations for policy and practice reflect the patient voice and patient priorities. Our approach to patient engagement is guided by the Authors and Consumers Together Impacting on eVidencE (ACTIVE) framework.^43^

### Selection criteria

The Sample, Phenomenon of Interest, Design, Evaluation and Research (SPIDER) framework will be used to define review search terms (table 1). In this review, we define MLTCs as the coexistence of two or more long-term physical and/or mental health conditions in an individual.^3^ We will include studies focusing on two or more of the following 11 conditions that were recommended in the existing literature as core to any systematic review of chronic disease^44^ as well as those included in the 2024 Health Survey for England^45^: hypertension, mental health conditions (such as depression and anxiety), type 2 diabetes, coronary heart disease, stroke, transient ischaemic attack, chronic obstructive pulmonary disease (COPD), cancer, heart failure, dementia and arthritis. These conditions are highly prevalent, often occur together in adults and account for a significant share of hospital activity and NHS expenditure in the UK.^23^ To be eligible for inclusion, all conditions described in the included studies must be diagnostically confirmed.

**Table 1:** Inclusion and exclusion criteria using the SPIDER framework

	Inclusion criteria	Exclusion criteria
Sample (S)	Studies addressing structures and processes of organising and delivering integrated care for adults aged ≥18 years with two or more of the following (diagnostically confirmed) MLTCs: hypertension, mental health conditions, type 2 diabetes, coronary heart disease, stroke, TIA, COPD, cancer, heart failure, dementia and arthritis. Studies conducted in high-income countries, as defined by the World Bank, using the country’s GNI per capita.^50^ Any study published between January 1990 and December 2025, reflecting the conceptualisation of integrated care in 1990 as a mechanism for organising healthcare internationally.^12^	Adults without MLTCs. Studies focusing on children. Mixed populations where it is not possible to separately extract and analyse data relating to adults. Studies focusing on one long-term chronic condition. Studies from low- and middle-income countries. Studies published before 1990.
Phenomenon of Interest (Pi)	Studies that examine the implementation of integrated care structures and processes in practice and the outcomes resulting from their use in adults with MLTCs. Any study presenting the structures and processes of implementing any form of integrated care service, models and/or configurations aimed at improving health outcomes for adults with MLTCs.^30^ Any study reporting the structures and processes of organising, coordinating and delivering integrated care across the three dimensions of the integrated health system: the macro (system-wide structures and policies), meso (organisational arrangements) and micro (clinical practice and patient-level interactions) levels.	Studies focusing solely on patient use or experiences of integrated care and/or MLTCs. Studies that focus solely on the management of a single condition. Studies describing hypothetical service design rather than real-world evaluation, implementation and outcomes.
Design (D)	Empirical studies, including all study designs.	Studies assessing the prevalence and epidemiology of MLTCs.
Evaluation (E)	Any study evaluating the organisation, design and implementation of integrated care models, structure and processes in managing individuals with MLTCs, alongside health and health service outcomes such as measuring improved quality of life and well-being, increased system efficiency, reduced healthcare utilisation, enhanced patient satisfaction, reduced health inequalities, better access and care management, improved communication and any other outcomes measured.	Evaluation of fragmented care, which typically addresses single disease management. The outcomes of single disease management.
Research type (R)	Empirical studies published in the English language. This is limited to the English language due to limited funding and human resources for professional translation of non-English records, and the research team’s language proficiency is in English.	Grey literature (eg, conference proceedings, dissertations, commentaries, editorials, theses and non-peer-reviewed articles). Systematic and non-systematic reviews (although citation searches will be conducted within these studies).

COPD, chronic obstructive pulmonary disease; GNI, gross national income; MLTC, multiple long-term conditions; SPIDER, Sample, Phenomenon of Interest, Design, Evaluation and Research; TIA, transient ischaemic attack.

### Search strategy and information sources

We will restrict our search to electronic medical literature databases (MEDLINE (Ovid), EMBASE (Ovid) and PsycINFO (Ovid)) to provide comprehensive, systematically indexed evidence on healthcare delivery and integrated care models. Given the clinical and healthcare service-focused nature of this review, these sources represent the most appropriate and efficient way to identify relevant evidence. A draft search strategy was developed by an information specialist (ND), with input from the research team (OT/JD/SD/AS), members of an expert panel that includes clinical experts in integrated care from the Midlands Patient Safety Research Collaboration (PSRC), local health service organisations and PPI members. Search terms included database-specific indexing terms where available. Free-text terms were developed with input from the information specialist (ND) and the research team (OT/JD/SD/AS) via an iterative process of scrutinising retrieved papers to inform further searching. The final search strategy will be developed in MEDLINE (Ovid) and adapted for other databases (online supplemental appendix 1). Other iterative searching techniques will also be employed, including citation searches of included articles and their authors and checking the reference lists of included articles (backwards citation searching). An updated search will be conducted across all electronic databases before the final reporting of the review to ensure inclusion of recently published studies to reflect current evidence.

We will not formally include grey literature, theoretical or policy literature, given that such sources may not provide empirical evidence of how organisational structures and processes for integrated care are implemented and experienced in practice. They may also focus on planned or hypothetical integrated care approaches or highlight structures and processes lacking adequate evaluation in real-world contexts. Where such literature returned by our searches may be deemed potentially relevant for context and interpreting our findings, we will flag this during screening, and a separate database of these papers will be retained. These citations will not be eligible for formal inclusion in the review. Still, their insights will be used to substantiate the review context and interpretation of findings in the final report.

### Study selection

The retrieved articles from the database searches will be exported to Covidence, a web application created for article screening^46^ and deduplication. Four reviewers (OT/JD/FB/SD) will independently double-screen titles/abstracts and full-text articles against the eligibility criteria to determine inclusion/exclusion (table 1). Disagreements will be resolved through discussion or, if necessary, by involving a fifth reviewer (AS). The process of study selection will be reported using the PRISMA flow chart template (online supplemental appendix 2).

### Quality assessment

The quality of the included articles will be independently assessed by two researchers (OT and FB), and any disagreements will be resolved by a third member of the research team (SD/AS). The methodological strengths and limitations of each included study will be assessed using the Mixed Methods Appraisal Tool (MMAT), a critical appraisal tool designed for reviews of qualitative, quantitative and mixed methods articles.^47^ Each study will be appraised across the relevant MMAT domain with responses recorded as ‘yes’, ‘no’ or ‘unsure’, in line with the tool’s guidance. No overall score will be calculated. Instead, the quality assessment of the studies will provide a structured narrative that highlights both the methodological strengths and limitations. No studies will be excluded based on quality score.

### Data extraction

Data extraction will be undertaken using Microsoft Forms. The draft form will be piloted on a random selection of included studies, with two reviewers independently extracting data and comparing the results to assess the consistency of the approach. The draft form will be finalised after piloting, with subsequent data extraction undertaken independently by three reviewers (OT/JD/FB). Discrepancies will be resolved by discussion or by consulting a third reviewer (SD/AS). We will systematically extract the following data items and information:

- Study characteristics (eg, study ID, title, author, year of publication and country).
- Study population (eg, age and sociodemographic characteristics) and combination of health conditions included (eg, type 2 diabetes, hypertension and COPD).
- Study aims and methodological approach (eg, qualitative, quantitative and mixed methods).
- Integrated care model organisation, structures and care processes (eg, how care is organised, coordinated and delivered).
- Conditions and settings that shape how the integrated care structures and processes operate in practice and the roles and care actors involved, including healthcare settings (eg, primary care and secondary care), organisational arrangements (eg, funding/commissioning arrangements, staffing models and leadership), processes of healthcare restructuring, type of organisation(s), healthcare providers, types of services, the name of the integrated care model and year of implementation (if applicable), care management functions, details of administrative oversight, funding processes and integrated care structures.
- Outcomes measured and reported in the included studies (including but not limited to): improved quality of life and well-being, increased system efficiency, reduced healthcare utilisation, enhanced patient satisfaction, reduced health inequalities, better access and care management and improved communication. Outcomes are expected to be measured using a mixture of patient-reported, service-level and system-level indicators and may be measured/reported using, for example, before-and-after or comparative study designs.
  This data will assist in examining how organisational arrangements are described and implemented in practice across different settings.

### Data synthesis

Given the likely heterogeneity of data, we will use a narrative approach to synthesis in two stages. In the first stage, after familiarisation with the data, we will seek to conceptualise the core components of the integrated care model organisation and delivery (structures and processes) for patients with MLTCs. Analysis will apply the RMIC framework (figure 1) to the data in order to categorise and interpret the data according to the mechanisms (horizontal and vertical integration) and levels (macro-meso-micro) of integrated care systems. Despite our focus on high-income countries, there may be substantial differences in the organisation and delivery of healthcare at the system level between countries. Summary information on the nature of the health systems within the countries represented within the evidence will be extracted, and any systems-level differences will be considered in our data synthesis. Similarly, for UK-based studies, the potential influence of major restructuring of healthcare systems over time (eg, the transition from Clinical Commissioning Groups to Integrated Care Systems) will be accounted for in our analysis. Where quantitative and qualitative data are included, data synthesis will use the Pillar Integration Process (PIP).^48 49^ This is a systematic approach for combining and presenting findings derived from multiple study designs. Findings will be merged across study designs (ie, qualitative themes and quantitative measures) to identify patterns and relationships (including concordance/discordance) and to present integrated results regardless of source data. The PIP findings will be mapped onto the appropriate RMIC level(s).

The first stage of the analysis will provide an evidence-based understanding of how integrated care models can be designed, organised and structured. Where data allow, a second stage of analysis/synthesis will focus on mapping the outcomes from integrated care onto the structures and processes derived from stage 1 to assess, where possible, the specific integrated care structures and processes that may be associated with specific outcomes. Outcome data are expected to be measured and reported using a mix of patient-reported, service-level and system-level indicators and may represent before-and-after or comparative analysis to assess outcome change over time or between groups.

### Relevance of evidence to the UK context

This review focuses on integrated service organisation and delivery, and the findings of the review will focus on the relevance to the UK health and care context. The Grading of Recommendations Assessment, Development and Evaluation (GRADE) and the GRADE–Confidence in the Evidence for Reviews of Qualitative Research will be used to guide evidence interpretation and presentation.

We will use GRADE assessment, focusing particularly on elements of (1) indirectness (applicability and whether the context of studies is comparable to UK settings, acknowledging differences and transferability), (2) consistency (understanding the variation reported across studies and settings and whether contextual or organisational factors can explain differences) and (3) methodological limitations (summarise common methodological concerns identified through the MMAT). This includes assessing whether the organisational structures and processes described in the study apply to UK settings, while acknowledging their differences and transferability. Overall, quality assessment will combine methodological evaluation with a structured consideration of applicability, providing a narrative that reflects both the rigour of the evidence and its usefulness for informing service delivery in the UK.

### Ethics and dissemination

This systematic review does not require approval from an ethics committee. The results will be disseminated in peer-reviewed journals and at international conferences, such as the International Conference on Integrated Care and the NHS Integrated Care System Conference, and at scientific meetings.

## Supplementary material

10.1136/bmjopen-2026-116356 online supplemental file 1

## Data Availability

Data sharing not applicable as no datasets generated and/or analysed for this study.
