# Supplementary material for: Evaluating the structure and process of effective integrated care for people with multiple long-term conditions: systematic review protocol
Source: BMJ Open. 2026 Jun 23;16(6):e116356. doi: 10.1136/bmjopen-2026-116356 (PMC13295912; doi:10.1136/bmjopen-2026-116356)
Supplement: online supplemental file 1 [file bmjopen-16-6-s001.docx]

**Supplementary Appendix 1: Full search strategy template (MEDLINE OVID)**

  Ovid MEDLINE(R) ALL

1 comorbidity/ or multimorbidity/ 137280

2 (Multimorbid* or multi-morbid* or comorbid* or co-morbid* or polymorbid* or poly-morbid* or multidisease* or multi-disease* or disease cluster* or LTCs or MLTCs).ti,ab,kf. 344248

3 1 or 2 414719

4 Chronic Disease/ 296124

5 exp diabetes mellitus/ or diabet*.ti,ab. 964040

6 exp hypertension/ or (hypertens* or "high blood pressure?").ti,ab. 645160

7 Coronary Disease/ or coronary heart disease?.ti,ab. 166708

8 exp Stroke/ or Ischemic Attack, Transient/ or exp Cerebral Hemorrhage/ or (stroke? or "cerebrovascular accident?").ti,ab. 458293

9 exp pulmonary disease chronic obstructive/ or (copd or (pulmonary adj2 (disease? or disorder?))).ti,ab. 128325

10 Parkinson Disease/ or Parkinson*.ti,ab. 176511

11 exp Mental Disorders/ or depression/ 1698755

12 ((Mental or mood or anxiety) adj (disorder? or disease?)).ti,ab. 123202

13 (Depression or bipolar or bi-polar or schizophren* or psychos*).ti,ab. 850151

14 exp Dementia/ or (dementia or alzheimer*).ti,ab. 371323

15 exp Renal Insufficiency, Chronic/ or ((Renal or kidney) adj (disease? or disorder? or insufficiency)).ti,ab. 293847

16 Heart Failure/ or heart failure, diastolic/ or heart failure, systolic/ or ventricular dysfunction/ or ((heart or myocardial) adj (failure or decompensation)).ti,ab. 291067

17 exp Gastrointestinal Diseases/ or (irritable bowel syndrome or inflammatory bowel disease or Crohn's or ulcerative colitis or coeliac disease or diverticular disease or stomach ulcer? or liver disease?).ti,ab. 1342791

18 exp Arthritis/ or (arthritis or osteoarthritis).ti,ab. 432619

19 exp asthma/ or asthma*.ti,ab. 218210

20 exp epilepsy/ or (epileps* or seizure?).ti,ab. 265631

21 exp Urological Manifestations/ or (urinary incontinence or lower urinary tract symptom?).ti,ab. 115099

22 exp Skin Diseases/ or (skin adj (disease? or disorder?)).ti,ab. 1333540

23 exp Ear Diseases/ or (deafness or tinnitus).ti,ab. or (ear adj (disorder? or disease?)).ti,ab. 198014

24 exp COVID-19/ or (post covid 19 condition? or long covid or Post-COVID-19 syndrome?).ti,ab. 311017

25 Frailty/ or frail*.ti,ab. 48146

26 or/4-25 8572428

27 ((Multipl* or concurrent* or coexist* or co-exist* or cooccur* or co-occur* or long-term or longterm or long-lasting or longstanding or chronic) adj3 (diseases or conditions or syndromes or disorders or illnesses)).ti,ab,kf. 242039

28 26 and 27 145913

29 3 or 28 [Multimorbidities] 541708

30 "delivery of health care, integrated"/ 15539

31 Intersectoral Collaboration/ 2899

32 (Integrat* or collaborat* or intersector* or inter-sector* or multisector* or multi-sector* or multi sector* or cross-sector* or joined up or managed continuity).ti,ab,kf. 1262107

33 (Multidisciplin* or multi-disciplin* or MDT? or multiinstitution* or multi-institution* or multiprofession* or multi-profession* or multisector* or multi-sector* or multispecialty or multi-specialit* or multihospital or multi-hospital or interdisciplin* or inter-disciplin* or inter-agency or interagency or interorgani?ational or inter-organi?ational or interinstitutional* or inter-institutional* or interprofessional* or inter-professional* or transdisciplin* or trans-disciplin* or trans disciplin* or co-manage* or comanage*).ti,ab,kf. 296440

34 ((Across or between or several or multiple) adj3 (networks or sectors or providers or teams or departments or services or institutions or disciplines or professions or specialities or specialisms)).ti,ab,kf. 82418

35 (System-wide or "whole system" or "whole-of-system" or networked).ti,ab,kf. 13960

36 Shared Medical Appointments/ 104

37 Health Care Coalitions/ 2375

38 ((Working or workforce) adj2 (together or partnership or coalition? or cooperat* or co-operat*)).ti,ab. 6275

39 Interdisciplinary Communication/ 18593

40 ((Joint* or joined or shared or holistic* or coordinat* or co-ordinat or blended or combin* or partnership or networked) adj3 (appointment* or service? or deliver* or health* or healthcare or work* or interven* or effort* or team* or pathway? or framework? or agencies or provider? or action or process* or communicat* or information or organi?ation* or decision*)).ti,ab,kf. 209456

41 Systems Integration/ or Multi-Institutional Systems/ 16613

42 electronic health records/ and (shared or interoperable or linked or longitudinal or unified).ti,ab. 3887

43 ((Shared or interoperable or joint or linked or longitudinal or unified) adj2 (health or healthcare) adj records).ti,ab,kf. 591

44 Hospital Shared Services/ 2159

45 (Physicians/ or General Practitioners/) and (exp Nurses/ or Pharmacists/ or Occupational Therapists/ or Physical Therapists/ or Psychotherapists/ or Psychologists/ or community health workers/ or Nutritionists/ or Allied Health Personnel/) 8853

46 Cooperative Behavior/ and (exp Health Personnel/ or Attitude of Health Personnel/ or Interprofessional Relations/ or Organizational Culture/) 13681

47 ((physician* or specialist* or consultant? or clinician?) and (nurse* or primary care provider* or general practitioner* or GP* or psychologist* or psychiatrist* or counse?lor* or pharmacist* or paraprofessional* or para-professional* or radiologist? or occupational therapist* or physical therapist? or physiotherapist? or nutritionist? or dietician? or allied health professional? or lay health worker* or community health worker?)).ti,ab,kf. 128195

48 or/30-47 [Integration] 1848811

49 models, organizational/ 19822

50 models, theoretical/ 168925

51 (Model$ adj1 (service? or leadership or deliver* or funding or manage* or organi?ation* or health-care or healthcare)).ti,ab,kf. 18731

52 "Organization and Administration"/ 14713

53 Decision Making, Organizational/ 11271

54 ((Organi?ation* or institution* or practice or "ways of working" or strategic* or context*) adj3 (structur* or factor? or determinant? or level? or cultur* or goal? or characteristic? or behavio?r* or decision?)).ti,ab,kf. 169696

55 leadership/ 52008

56 ((Clinical or healthcare or health-care or organi?ation* or operational) adj2 leadership).ti,ab,kf. 4619

57 ((Model$ or level?) adj2 (vertical or horizontal or macro or micro or meso)).ti,ab,kf. 11287

58 strategic planning/ or health planning/ 22395

59 ((Planning or manag*) adj1 (resource? or health or healthcare or service?)).ti,ab,kf. 53471

60 ((organi?ation* or funding or commission* or staff* or work* or financ*) adj5 (arrange* or process*)).ti,ab,kf. 61528

61 Delivery of Health Care/ 126727

62 (Deliver* adj1 (health-care or healthcare or service?)).ti,ab,kf. 53721

63 Efficiency, Organizational/ 22985

64 ((Organi?ation* or operational or service? or program* or health-care or healthcare or program*) adj3 (improve* or quality or efficien* or impact* or benefi*)).ti,ab,kf. 218952

65 Implementation Science/ or Health Plan Implementation/ 8648

66 ((Organi?ation* or operational or service? or program* or health-care or healthcare or program* or policy or policies) adj3 implement*).ti,ab,kf. 77026

67 Organizational Innovation/ 25686

68 ((Organi?ation* or institution* or practice or ways of working or strateg* or operation* or service? or program* or health-care or healthcare) adj3 (innovat* or change? or develop* or transform* or re-structur* or restructur* or scale* up)).ti,ab,kf. 368975

69 ((Organi?ation* or operational or service? or program* or health-care or healthcare or program* or profession*) adj2 (leadership? or governance)).ti,ab,kf. 7577

70 patient satisfaction/ or patient preference/ or Patient-Centered Care/ or Professional-Patient Relations/ 158933

71 ((patient-centred or patient-centered) adj2 (care or manage$ or program$ or service$ or policy or policies)).ti,ab,kf. 19634

72 (patient? satisf* or patient? pref*).ti,ab,kf. 90035

73 or/49-72 [Organisational structures] 1462962

74 afghanistan/ or africa/ or africa, northern/ or africa, central/ or africa, eastern/ or "africa south of the sahara"/ or africa, southern/ or africa, western/ or albania/ or algeria/ or andorra/ or angola/ or "antigua and barbuda"/ or argentina/ or armenia/ or azerbaijan/ or bahamas/ or bahrain/ or bangladesh/ or barbados/ or belize/ or benin/ or bhutan/ or bolivia/ or borneo/ or "bosnia and herzegovina"/ or botswana/ or brazil/ or brunei/ or bulgaria/ or burkina faso/ or burundi/ or cabo verde/ or cambodia/ or cameroon/ or central african republic/ or chad/ or exp china/ or comoros/ or congo/ or cote d'ivoire/ or croatia/ or cuba/ or "democratic republic of the congo"/ or cyprus/ or djibouti/ or dominica/ or dominican republic/ or ecuador/ or egypt/ or el salvador/ or equatorial guinea/ or eritrea/ or eswatini/ or ethiopia/ or fiji/ or gabon/ or gambia/ or "georgia (republic)"/ or ghana/ or grenada/ or guatemala/ or guinea/ or guinea-bissau/ or guyana/ or haiti/ or honduras/ or independent state of samoa/ or exp india/ or indian ocean islands/ or indochina/ or indonesia/ or iran/ or iraq/ or jamaica/ or jordan/ or kazakhstan/ or kenya/ or kosovo/ or kuwait/ or kyrgyzstan/ or laos/ or lebanon/ or liechtenstein/ or lesotho/ or liberia/ or libya/ or madagascar/ or malaysia/ or malawi/ or mali/ or malta/ or mauritania/ or mauritius/ or mekong valley/ or melanesia/ or micronesia/ or monaco/ or mongolia/ or montenegro/ or morocco/ or mozambique/ or myanmar/ or namibia/ or nepal/ or nicaragua/ or niger/ or nigeria/ or oman/ or pakistan/ or palau/ or exp panama/ or papua new guinea/ or paraguay/ or peru/ or philippines/ or qatar/ or "republic of belarus"/ or "republic of north macedonia"/ or romania/ or exp russia/ or rwanda/ or "saint kitts and nevis"/ or saint lucia/ or "saint vincent and the grenadines"/ or "sao tome and principe"/ or saudi arabia/ or serbia/ or sierra leone/ or senegal/ or seychelles/ or singapore/ or somalia/ or south africa/ or south sudan/ or sri lanka/ or sudan/ or suriname/ or syria/ or taiwan/ or tajikistan/ or tanzania/ or thailand/ or timor-leste/ or togo/ or tonga/ or "trinidad and tobago"/ or tunisia/ or turkmenistan/ or uganda/ or ukraine/ or united arab emirates/ or uruguay/ or uzbekistan/ or vanuatu/ or venezuela/ or vietnam/ or west indies/ or yemen/ or zambia/ or zimbabwe/ 1483833

75 "Organisation for Economic Co-Operation and Development"/ 728

76 australasia/ or exp australia/ or austria/ or baltic states/ or belgium/ or exp canada/ or chile/ or colombia/ or costa rica/ or czech republic/ or exp denmark/ or estonia/ or europe/ or finland/ or exp france/ or exp germany/ or greece/ or hungary/ or iceland/ or ireland/ or israel/ or exp italy/ or exp japan/ or korea/ or latvia/ or lithuania/ or luxembourg/ or mexico/ or netherlands/ or new zealand/ or north america/ or exp norway/ or poland/ or portugal/ or exp "republic of korea"/ or "scandinavian and nordic countries"/ or slovakia/ or slovenia/ or spain/ or sweden/ or switzerland/ or turkey/ or exp united kingdom/ or exp united states/ 3743274

77 European Union/ 18834

78 Developed Countries/ 21969

79 or/75-78 3760497

80 74 not 79 1389037

81 Editorial/ 746875

82 Comment/ 1060385

83 exp animals/ not humans/ 5418381

84 or/80-83 8177139

85 29 and 48 and 73 10515

86 85 not 84 9743

87 limit 86 to ed=19900101-20260127 8339

88 limit 86 to ep=19900101-20260127 6726

89 limit 86 to ez=19900101-20260127 9727

90 limit 86 to dt=19900101-20260127 9727

91 87 or 88 or 89 or 90 9727

92 limit 91 to english language 9241

**Supplementary Appendix 2: The Preferred Reporting Items for Systematic Reviews and Meta-Analyses(PRISMA) flow chart template^47^**

**Identification of studies via databases and registers**

**Identification of studies via other methods**

Records identified from*:

Databases (n = )

Registers (n = )

Records removed *before screening*:

Duplicate records removed (n = )

Records marked as ineligible by automation tools (n = )

Records removed for other reasons (n = )

Records identified from:

Websites (n = )

Organisations (n = )

Citation searching (n = )

etc.

**Identification**

Records screened

(n = )

Records excluded**

(n = )

**Screening**

Reports sought for retrieval

(n = )

Reports not retrieved

(n = )

Reports sought for retrieval

(n = )

Reports not retrieved

(n = )

Reports assessed for eligibility

(n = )

Reports excluded:

Reason 1 (n = )

Reason 2 (n = )

Reason 3 (n = )

etc.

Reports assessed for eligibility

(n = )

Reports excluded:

Reason 1 (n = )

Reason 2 (n = )

Reason 3 (n = )

etc.

**Included**

Studies included in review

(n = )

Reports of included studies

(n = )

## **Supplementary Appendix 3: PRISMA-P (Preferred Reporting Items for Systematic Review and Meta-Analysis Protocols) 2015 checklist^41^**

| Section and topic | Item No | Checklist item |  |
| --- | --- | --- | --- |
| ADMINISTRATIVE INFORMATION | | |  |
| Title: |  |  |  |
| Identification | 1a | Identify the report as a protocol of a systematic review | Title |
| Update | 1b | If the protocol is for an update of a previous systematic review, identify as such | Not Applicable |
| Registration | 2 | If registered, provide the name of the registry (such as PROSPERO) and registration number | **PROSPERO registration:** CRD420251143298. |
| Authors: |  |  |  |
| Contact | 3a | Provide name, institutional affiliation, e-mail address of all protocol authors; provide physical mailing address of corresponding author | Title Page, Line 10 – 14 |
| Contributions | 3b | Describe contributions of protocol authors and identify the guarantor of the review | Contributors section Line 338- 342 |
| Amendments | 4 | If the protocol represents an amendment of a previously completed or published protocol, identify as such and list changes; otherwise, state plan for documenting important protocol amendments | Not applicable |
| Support: |  |  |  |
| Sources | 5a | Indicate sources of financial or other support for the review | Funding statement, Line 346 - 352 |
| Sponsor | 5b | Provide name for the review funder and/or sponsor | Funding statement, Line 346 - 352 |
| Role of sponsor or funder | 5c | Describe roles of funder(s), sponsor(s), and/or institution(s), if any, in developing the protocol | Funding statement, Line 346 - 352 |
| INTRODUCTION | | |  |
| Rationale | 6 | Describe the rationale for the review in the context of what is already known | Introduction section, Line 90 - 141 |
| Objectives | 7 | Provide an explicit statement of the question(s) the review will address with reference to participants, interventions, comparators, and outcomes (PICO) | Line 146 - 150 and Table 1 Line 207 -208 |
| METHODS | | |  |
| Eligibility criteria | 8 | Specify the study characteristics (such as PICO, study design, setting, time frame) and report characteristics (such as years considered, language, publication status) to be used as criteria for eligibility for the review | Table 1, Line 207 -208 |
| Information sources | 9 | Describe all intended information sources (such as electronic databases, contact with study authors, trial registers or other grey literature sources) with planned dates of coverage | Line 210 – 211 |
| Search strategy | 10 | Present draft of search strategy to be used for at least one electronic database, including planned limits, such that it could be repeated | Appendix 1 |
| Study records: |  |  |  |
| Data management | 11a | Describe the mechanism(s) that will be used to manage records and data throughout the review | Line 236 |
| Selection process | 11b | State the process that will be used for selecting studies (such as two independent reviewers) through each phase of the review (that is, screening, eligibility and inclusion in meta-analysis) | Line 236 – 242 |
| Data collection process | 11c | Describe planned method of extracting data from reports (such as piloting forms, done independently, in duplicate), any processes for obtaining and confirming data from investigators | Line 253 - 280 |
| Data items | 12 | List and define all variables for which data will be sought (such as PICO items, funding sources), any pre-planned data assumptions and simplifications | Line 260– 280 |
| Outcomes and prioritisation | 13 | List and define all outcomes for which data will be sought, including prioritisation of main and additional outcomes, with rationale | Line 260– 280 |
| Risk of bias in individual studies | 14 | Describe anticipated methods for assessing risk of bias of individual studies, including whether this will be done at the outcome or study level, or both; state how this information will be used in data synthesis | Line 243 -252 |
| Data synthesis | 15a | Describe criteria under which study data will be quantitatively synthesised | Line 281 – 298 |
|  | 15b | If data are appropriate for quantitative synthesis, describe planned summary measures, methods of handling data and methods of combining data from studies, including any planned exploration of consistency (such as I^2^, Kendall’s τ) | Not applicable |
|  | 15c | Describe any proposed additional analyses (such as sensitivity or subgroup analyses, meta-regression) | Line 281 – 298 |
|  | 15d | If quantitative synthesis is not appropriate, describe the type of summary planned | Line 281 – 298 |
| Meta-bias(es) | 16 | Specify any planned assessment of meta-bias(es) (such as publication bias across studies, selective reporting within studies) | Not Applicable |
| Confidence in cumulative evidence | 17 | Describe how the strength of the body of evidence will be assessed (such as GRADE) | Line 309 -324 |
