# Supplementary material for: Evaluating the structure and process of effective integrated care for people with multiple long-term conditions: systematic review protocol
Source: BMJ Open. 2026 Jun 23;16(6):e116356. doi: 10.1136/bmjopen-2026-116356 (PMC13295912; doi:10.1136/bmjopen-2026-116356)
Supplement: Supplementary file 2 [file bmjopen-2026-116356.reviewer_comments.pdf]

PEER REVIEW HISTORY

BMJ Open publishes all reviews undertaken for accepted manuscripts. Reviewers are asked to complete a checklist review form and are provided with free text boxes to elaborate on their assessment. These free text comments are reproduced below.

ARTICLE DETAILS

Title (Provisional)

Evaluating the structure and process of effective integrated care for people with multiple long-term conditions -systematic review protocol

Authors

Todowede, Olamide; Damery, Sarah; Dracup, Naila; Donoghue, Jeremiah; Butt, Furqan; Grove, Amy; Scantlebury, Arabella

---

VERSION 1 - REVIEW

**Reviewer**	**1**
**Name**	**Lee, Poay Sian Sabrina**
**Affiliation**	**National Healthcare Group Polyclinics, Clinical Research Unit**
**Date**	**06-Feb-2026**
**COI**	**None**

Thank you for the opportunity to review this systematic review protocol.

Overall excellent piece of writing.

Minor comments:

1. There was no explanation of why including 1990 as the beginning of the search date in the Introduction. It was only apparent in the Methods in line 55. Would have been better if that piece of information was included in the Introduction for clarity.

2. There was a typo in line 227 for EndNote

3. Understandbly that this systematic review was planned to inform policy for the UK, hence to include high income countries only. I am trying to see what value this would add to the global literature, as integrated care is a huge issue in the lower middle countries as well. Would like to hear the thoughts from the authors for this point.

**Reviewer**	**2**
**Name**	**Dregan, Alexandru**

Affiliation **King's College London, UK, Primary Care and Public Health Sciences**

Date **07-Mar-2026**

COI **None**

This is a well-written manuscript detailing the plans for a future systematic review on the effectiveness of integrated care for patients with multimorbidity. This Reviewer has several comments that authors may wish to consider.

1. Unclear to this Reviewer the decision to focus on 11 long-term conditions, specifically why referencing HSE as a rationale for the selection? Why not use the Delphi exercise published by Ho et al., 2012?

2. When were integrated care services implemented in different contexts/countries (eg ICBs were set up in 2022 in the UK)? Shouldn't the period be informed by such timing?

3. Regarding the Design exclusion criteria - are epidemiological studies that compared before and after excluded from the review? Surely they should be included

4. On line 233, suppose it is meant to say a fourth reviewer ?

5. What if the evidence fails to uncover any effective integrated systems? Please see the 3D trial (Salisbury et al., 2018).

6. What are the research priorities that the review aims to address?

VERSION 1 - AUTHOR RESPONSE

**Reviewer: 1**<br>**Dr Poay Sian Sabrina Lee, National Healthcare Group Polyclinics**		
**S/N**	**Reviewers Comments**	**Authors Response**
1	There was no explanation of why including 1990 as the beginning of the search date in the Introduction. It was only apparent in the Methods in line 55. Would have been better if that piece of information was included in the Introduction for clarity.	Thank you for your comment. We have now moved the explanation of the Integrated care conceptualisation from the methods to the Introduction section. This now reads:<br>“In the 1990s, integrated care was conceptualised as a concept of organising healthcare by focusing on the needs of patients or populations and addressing the adverse outcomes and experiences of care linked to a

		fragmented healthcare system[12]. " Refer to Paragraph 3 of the introduction section
2	There was a typo in line 227 for EndNote	Thank you for the comment. All endnotes have been checked for errors and corrected
3	Understandably that this systematic review was planned to inform policy for the UK, hence to include high income countries only. I am trying to see what value this would add to the global literature, as integrated care is a huge issue in the lower middle countries as well. Would like to hear the thoughts from the authors for this point.	Thank you for this important observation. We agree that integrated care is a critical issue globally, including in low- and middle-income countries, where health system challenges and the burden of multimorbidity are substantial. However, this review was commissioned by the UK NIHR Evidence Synthesis Programme by the National Institute for Health and Care Research (NIHR) with the explicit goal of drawing on evidence from healthcare contexts comparable to and potentially generalisable to the UK. This was to allow the review results to inform UK integrated care policy and service organisation/delivery. For this reason, we restricted inclusion to high-income country settings, where health system structures, financing mechanisms, and service organisation are more comparable, thereby enhancing the applicability and transferability of findings to the UK context. This is outlined in the manuscript's introduction section (Lines 129–142). We have not made any changes to the manuscript based on this comment.
**Reviewer: 2**<br>**Dr Alexandru Dregan, King's College London, UK**		
1	Unclear to this Reviewer the decision to focus on 11 long-term conditions, specifically why referencing HSE as a rationale for the selection? Why not use the	Thank you for this point. We assume that the reviewer is referring to the Ho et al (2022) paper published in BMJ Medicine rather than a paper by Ho et al (2012). We were aware of this publication when planning the review. As the

	Delphi exercise published by Ho et al., 2012?	authors of that study argue, "the choice of conditions to include in measurement should be sensitive to purpose and local context". All of the conditions we chose to include in our review are also represented in the list covered by Ho et al (2022), and so this reference would not substantially change our eligibility criteria. We have the additional goal of understanding health service organisation in relation to the conditions that may be most burdensome to individuals, account for the greatest costs to health services, have the greatest impact on quality of life, and have a high enough prevalence that explicit integrated care services/interventions will have been developed to assess care quality/effectiveness for these conditions.<br>We also asked PPI representatives and other stakeholders (including clinical experts) at the design and planning stage for their views about which conditions we should include in the review, and our choice of conditions also reflects the outcome of these discussions.<br><br>Our specific reasons for the inclusion of the 11 chosen conditions are given in the manuscript and we have not made any changes following this comment other than to explicitly acknowledge the role of the PPI contributors in helping us to shape the choices of conditions we made (PPI section).
2	When were integrated care services implemented in different contexts/countries (eg ICBs were	Thank you for this comment. Integrated care as a means of organising and delivering health services predates the formation of Integrated

	set up in 2022 in the UK)? Shouldn't the period be informed by such timing?	Care Boards in the UK by at least three decades, with integration having been described using multiple terminologies since the 1990s. Successive organisational structures at the systems level (e.g. Clinical Commissioning Groups, Primary Care Trusts) may be relevant structural contributors to care integration and we are aware of the current re-structuring of ICBs and NHS England that is currently underway and will provide an interesting backdrop within which to discuss and disseminate our findings when they are available.<br>Other high-income countries will have their own changes to definitions and service organisation over time, and we will take into account the potential impact/influence of longitudinal changes within the UK (and other) contexts when characterising the evidence within our analysis. We have added some text to the analysis section to make this clear. Line 296 – 298 “synthesis. Similarly, for UK-based studies, the potential influence of major restructuring of healthcare systems over time (e.g. the transition from Clinical Commissioning Groups to Integrated Care Systems) will be accounted for in our analysis and will undoubtedly influence our discussion and methods of dissemination.
3	Regarding the Design exclusion criteria - are epidemiological studies that compared before and after excluded from the review? Surely they should be included	Thank you for the comment. We haven't excluded any empirical studies based on methods, and this has been stated in the Manuscript. Table 1 Line 207 – 208 Design section, “Empirical studies, including all study designs”. Before-after studies, or those reporting

		quality improvement initiatives (for example) will be eligible for inclusion.
4	On line 233, suppose it is meant to say a fourth reviewer ?	Thank you, this has been changed to the fifth reviewer, Line 239 – 240: “Disagreements will be resolved through discussion or, if necessary, by involving a fourth reviewer (AS).”
5	What if the evidence fails to uncover any effective integrated systems? Please see the 3D trial (Salisbury et al., 2018).	Thank you for the comment. Our review is not explicitly focused on identifying effective integrated care systems (although indicators of effectiveness or success will be extracted for our analysis). We focus instead on the structures, processes and key operational features that may be in place to facilitate integrated care organisation and delivery. We are aware that integration is not always effective even in ‘ideal conditions’, and in focusing on the structures and processes that may facilitate integrated care at the macro, meso and micro scale, we hope to draw out these nuances about the relevant levers of change.<br>It is also likely that ‘effectiveness’ is not merely a presence/absence condition – interventions or approaches that are effective in one regard (e.g. in terms of patient satisfaction) may be ‘ineffective’ in others (e.g. cost of model implementation). Thus, effectiveness is multifaceted in this context. We have not made any changes to the manuscript following this comment
6	What are the research priorities that the review aims to address?	Thank you for your comment. This has been stated in the introduction section, lines 137 – 142: “ This review will provide evidence to inform current global and UK health policy. Notably, this review will provide evidence that addresses

		the current UK government's long-term plan and could be used to optimise service delivery and reduce resource use. The review also addresses research priority areas, including multiple evidence gaps outlined in the NIHR Strategic Framework for Multiple Long-Term Conditions[37] and will provide direct evidence to inform numerous current global and national policy priority areas[17]. "

VERSION 2 - REVIEW

Reviewer 2

Name Dregan, Alexandru

Affiliation King's College London, UK, Primary Care and Public Health Sciences

Date 09-May-2026

COI

This Reviewer is satisfied with the authors responses, and has no further comments. Thank you.
